# Sphingosylphosphorylcholine potentiates vasoreactivity and voltage-gated Ca^2+^ entry via NOX1 and reactive oxygen species

**DOI:** 10.1093/cvr/cvv029

**Published:** 2015-02-06

**Authors:** Yasin Shaifta, Vladimir A. Snetkov, Jesus Prieto-Lloret, Greg A. Knock, Sergey V. Smirnov, Philip I. Aaronson, Jeremy P.T. Ward

**Affiliations:** 1 Division of Asthma, Allergy, and Lung Biology, King's College London, 5th Floor Tower Wing, Guy's Campus, London SE1 9RT, UK; 2 Department of Pharmacy and Pharmacology, University of Bath, Bath, UK

**Keywords:** NADPH oxidase, Vascular smooth muscle, L-type Ca^2+^ channels, Protein kinase C epsilon, Reactive oxygen species

## Abstract

**Aims:**

Sphingosylphosphorylcholine (SPC) elicits vasoconstriction at micromolar concentrations. At lower concentrations (≤1 µmol/L), however, it does not constrict intrapulmonary arteries (IPAs), but strongly potentiates vasoreactivity. Our aim was to determine whether this also occurs in a systemic artery and to delineate the signalling pathway.

**Methods and results:**

Rat mesenteric arteries and IPAs mounted on a myograph were challenged with ∼25 mmol/L [K^+^] to induce a small vasoconstriction. SPC (1 µmol/L) dramatically potentiated this constriction in all arteries by ∼400%. The potentiation was greatly suppressed or abolished by inhibition of phospholipase C (PLC; U73122), PKCε (inhibitory peptide), Src (PP2), and NADPH oxidase (VAS2870), and also by Tempol (superoxide scavenger), but not by inhibition of Rho kinase (Y27632). Potentiation was lost in mesenteric arteries from p47^phox–/–^, but not NOX2^−/–^, mice. The intracellular superoxide generator LY83583 mimicked the effect of SPC. SPC elevated reactive oxygen species (ROS) in vascular smooth muscle cells, and this was blocked by PP2, VAS2870, and siRNA knockdown of PKCε. SPC (1 µmol/L) significantly reduced the EC_50_ for U46619-induced vasoconstriction, an action ablated by Tempol. In patch-clamped mesenteric artery cells, SPC (200 nmol/L) enhanced Ba^2+^ current through L-type Ca^2+^ channels, an action abolished by Tempol but mimicked by LY83583.

**Conclusion:**

Our results suggest that low concentrations of SPC activate a PLC-coupled and NOX1-mediated increase in ROS, with consequent enhancement of voltage-gated Ca^2+^ entry and thus vasoreactivity. We speculate that this pathway is not specific for SPC, but may also contribute to vasoconstriction elicited by other G-protein coupled receptor and PLC-coupled agonists.

## 1. Introduction

Sphingosylphosphorylcholine (SPC) is derived from membrane sphingomyelin and is present in the plasma in submicromolar-free concentrations and as a major component of low- and high-density lipoproteins; it is also released from activated platelets.^1–3^ Sphingolipids including SPC and sphingosine-1-phosphate (S1P) have been associated with cardiovascular disease, but whereas S1P has been extensively investigated and its receptors cloned, no specific receptor for SPC has been positively identified and there are no selective pharmacological antagonists. The actions of SPC are, however, stereospecific and dependent on phospholipase C (PLC), suggesting that they are mediated via a G-protein coupled receptor.^1,4,5^

SPC induces vasoconstriction in isolated arteries via activation of Ca^2+^ entry and Rho kinase-mediated Ca^2+^-sensitization, with an EC_50_ of ∼12 µmol/L.^1,5–9^ However, plasma concentrations are at least 10-fold less than this, raising questions concerning the physiological relevance of vasoconstriction induced by SPC. Conversely, we have demonstrated in rat intrapulmonary arteries (IPAs) that although low concentrations (1 µmol/L or less) of SPC do not on their own elicit vasoconstriction, cause depolarization, elevate [Ca^2+^]_i_, or activate Rho kinase, they strongly potentiate vasoreactivity by enhancing Ca^2+^ entry induced by other stimuli.^10^ Although this effect was also stereospecific and dependent on PLC,^10^ it therefore clearly differs from the mechanisms previously shown to underlie vasoconstriction induced by higher concentrations of SPC. This implies an additional and hitherto unrecognized high affinity signalling pathway that could be of physiological relevance.

SPC has been reported to induce generation of reactive oxygen species (ROS) in a variety of non-muscle cell types, probably via NADPH oxidase (NOX),^11–14^ and ROS have been shown to enhance Ca^2+^ entry through L-type voltage-gated Ca^2+^ channels in vascular smooth muscle and cardiac myocytes.^15–18^ ROS have also been implicated in signalling pathways initiated by other PLC-coupled vasoconstrictor agonists, including angiotensin II and endothelin.^16,17,19^ This led us to hypothesize that a NOX/ROS-mediated pathway might underlie the SPC-induced enhancement of IPA vasoreactivity. As the pulmonary vasculature exhibits some unique responses to changes in redox state and ROS,^20,21^ we focused here on mesenteric artery (MA) to determine whether our previous observations were specific to the pulmonary vasculature. We show that low, subcontractile concentrations of SPC potentiate vasoreactivity of both MA and IPA through the same pathway, which involves PLC- and PKCε-dependent activation of NOX1, increased production of ROS, and consequent enhancement of Ca^2+^ entry via L-type channels.

## 2. Methods

### 2.1 Animals and tissues

The study conforms with UK Home Office regulations and Directive 2010/63/EU of the European Parliament. Adult male Wistar rats were killed by a lethal overdose of pentobarbital (i.p.). The lungs, mesentery, and in some cases sections of small renal or main femoral artery were excised and placed in cold physiological saline solution (PSS; in mmol/L: 118 NaCl, 24 NaHCO_3_, 1 MgSO_4_, 0.44 NaH_2_PO_4_, 4 KCl, 5.5 glucose, and 1.8 CaCl_2_). Male mice (6–8 weeks old) lacking genes for gp91*^phox^*^22^ (NOX2), p47*^phox^* ^23^ (background for both C57BL/6) or PKCδ (background 129/SV)^24^, or matched wild-type (WT), were killed by a Home Office approved method, the mesentery removed and placed in cold PSS.

Small IPA, MA, renal artery (200–500 µm i.d.) or femoral artery segments (1–2 mm i.d.) were dissected free of connective tissue, mounted on a myograph (Danish Myo Technology, Denmark), and bathed in PSS gassed with 5% CO_2_, balance air (pH 7.4) at 37°C. Vessels were stretched to equivalent transmural pressures of ∼25 (IPA) or ∼90 mmHg (MA and renal), and pre-conditioned by repeated exposure to 80 mmol/L K^+^ PSS (KPSS, equimolar substitution for NaCl) as previously described.^10,20^ Experiments were performed after ∼30 min to allow for stabilization. Tension was recorded using Acquisition Engine software (Cairn Research Ltd, Faversham, UK).

### 2.2 Cell culture, siRNA design, and cell transfection

Pulmonary artery smooth muscle cells (PASMCs) were dispersed from IPA using collagenase (type XI, 2 mg/mL) and papain (1 mg/mL), and cultured in DMEM containing 10% FCS as previously described.^10^ PASMCs from passages 3–4 were growth-arrested in serum-free medium for 24 h before use; each cell line was verified as smooth muscle by immunostaining for smooth muscle α-actin, calponin, and desmin (Sigma-Aldrich, Poole, UK).

siRNAs were designed as described previously.^25^ The 19-nucleotide target sequences (PKCδ-siRNA: position 883–901, GenBank accession no. BC076505; PKCε-siRNA: position 2079–2097, GenBank accession no. AY642593) were synthesized into 64–65 mer oligonucleotides with BamHI/HindIII overhangs (Sigma-Aldrich) and cloned into the expression vector pSilencer 3.0-H1 (Life Technologies Ltd, Paisley, UK). All clones were purified using an EndoFree Plasmid Maxi Kit (Qiagen Ltd, Crawley, UK) and sequenced (Geneservice Ltd, Cambridge, UK). PASMCs were transfected using the Basic Nucleofector™ Kit for primary mammalian smooth muscle cells and a nucleofector device (Nucleofector™ Technology, Lonza, Slough, UK); after 72 h cells were serum starved for 24 h prior to use. Transfection efficiency was >80%, as determined using pmaxGFP (green fluorescent protein-expressing vector) provided in the kit and confirmed by fluorescence microscopy. Efficiency and selectivity of knockdown was confirmed by western blot.

### 2.3 Estimation of ROS

As a qualitative, real-time estimation in intact arteries maintained under identical conditions to contraction studies, MAs were mounted on a confocal wire myograph (Danish Myo Technology) and pre-conditioned as above. Following incubation with 10 µmol/L carboxy 2′,7′-dichlorofluorescin–diacetate (C-DCFH/DA) for 45 min at 37°C, excess dye was washed off and tissue fluorescence of oxidized C-DCF (excitation 490 nm and emission 530 nm) recorded every 30 s using an inverted microscope (Zeiss UK Ltd) and microfluorimeter (Cairn Research Ltd). After a stable baseline was established (∼30 min), SPC was added to the bath.

ROS generation in cultured cells was estimated using lucigenin-enhanced luminescence. PASMCs (passage 4) were cultured to confluence in 24-well plates and growth-arrested for 24 h. Medium was replaced with gassed PSS at 37°C containing 5 µmol/L lucigenin and 100 µmol/L NADPH, to which SPC and pharmacological inhibitors were added. Luminescence was measured at 37°C using a Hidex Chameleon plate reader (Hidex, Finland).

### 2.4 Electrophysiology

Freshly isolated MA smooth muscle cells (MASMCs) were obtained from third- to fourth-order MA by enzymatic dispersion, and recordings of whole-cell currents performed with patch clamp as described previously.^26^ Ba^2+^ was used as a charge carrier to record currents (*I*_Ba_) through voltage-gated L-type Ca^2+^ channels, with an extracellular solution containing (mmol/L): 10 BaCl_2_, 130 NaCl, 5 CsCl, 1 MgCl_2_, 5 HEPES, 5 glucose, pH 7.35 and a pipette solution containing (mmol/L): 120 Cs methansulfonate, 20 CsCl, 2 MgATP, 0.5 Na_2_GTP, 0.3 MgCl_2_ and 5 HEPES, pH 7.2. Cells were equilibrated with pipette solution for 3 min after whole-cell access before recording the control (time 0) current–voltage (*I*–*V*) relationship using 120 ms voltage steps between −80 and +80 mV; holding potential was −70 mV. Cells were then incubated for 5 min with either 200 nmol/L SPC, 1 µmol/L LY83583, or in the absence of drug (time control), prior to recording of the test *I*–*V* relationship. The effect of Tempol (3 mmol/L) was studied on cells preincubated for 2 min before addition of 200 nmol/L SPC.

### 2.5 Calculations and statistical analysis

Tension was normalized to the response to KPSS, or for potentiation experiments to the control response prior to addition of SPC. Results are expressed as means ± SEM. Concentration–response curves were fitted to individual experiments using a Hill equation to provide EC_50_ and fitted maximum (*V*_max_; Sigmaplot 12, Systat Software Inc., CA, USA); for analysis EC_50_ was expressed as pD_2_ (−log EC_50_). Statistical analysis was performed using ANOVA with a Holm-Sidak *post hoc* unless otherwise stated (Sigmaplot, Systat Software Inc.). Statistical significance was deemed if *P* < 0.05.

### 2.6 Reagents

U73122, Gö6983, Gö6976, PP2, and rottlerin were obtained from Calbiochem, UK; C-DCFH/DA from Invitrogen, UK, and all other reagents including PKCε translocation inhibitor peptide from Sigma-Aldrich.

## 3. Results

### 3.1 Potentiation of vasoconstriction by subcontractile concentrations of SPC

**Figure 1:**
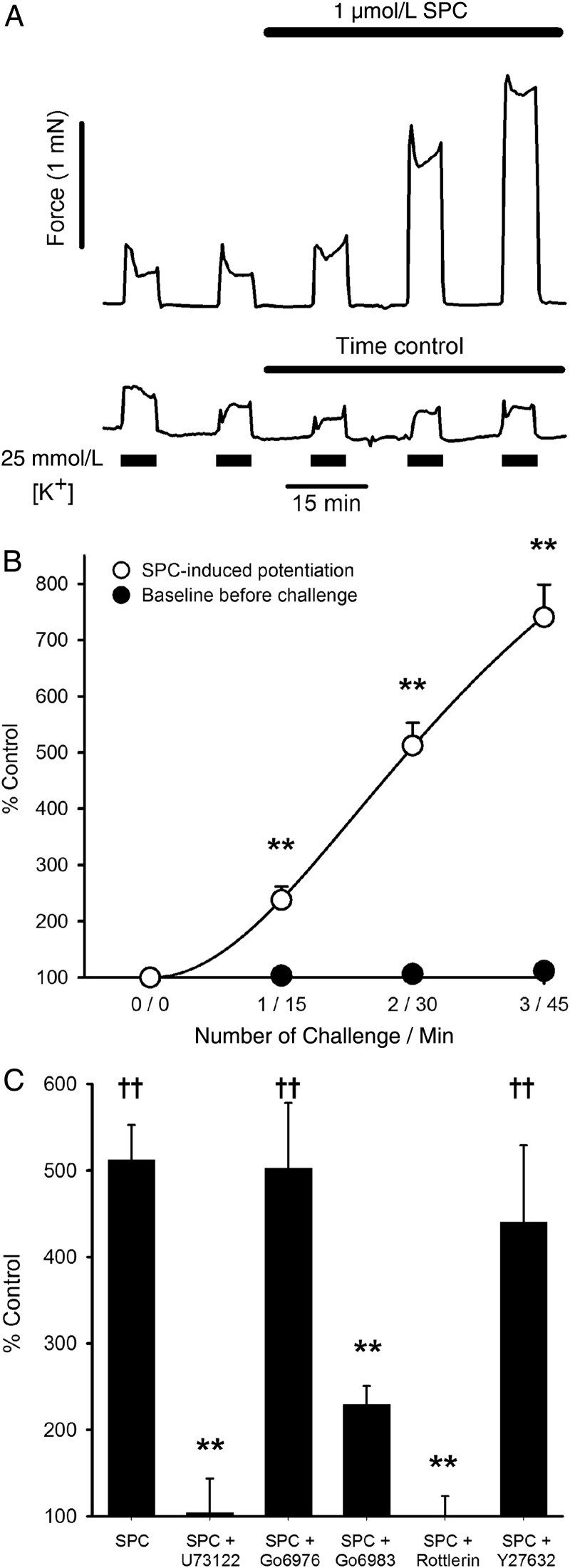
SPC-induced potentiation of tension development in MA. (*A*) Typical recordings of tension developed in rat MA to 5 min challenges with PSS containing 25 mmol/L [K^+^], demonstrating strong potentiation of contraction following addition of 1 µmol/L SPC compared with time control. (*B*) Mean data from 31 MAs (24 rats). Open symbols represent an increase in tension over control response (challenge 0 at time 0, i.e. 0 / 0) following addition of SPC. Filled symbols represent tension immediately preceding each depolarizing challenge, demonstrating a stable baseline. Bars = SEM, where not shown, smaller than symbol. ** *P* < 0.001 vs. control; repeated measures (RM) ANOVA on ranks, Tukey *post hoc*. (*C*) SPC-induced potentiation in MA from 24 rats at 30 min (challenge 2) in the presence of U73122 (PLC inhibitor, *n* = 4), Go6976 (conventional PKC inhibitor, *n* = 7), Go6983 (broad-spectrum PKC inhibitor, *n* = 6), rottlerin (putative PKCδ inhibitor, *n* = 5), and Y27362 (Rho kinase inhibitor, *n* = 7). Bars = SEM. **P < 0.001 vs. SPC alone; **^††^***P* < 0.001 vs. control; two-way ANOVA, Holm-Sidak *post hoc*.

**Figure 2:**
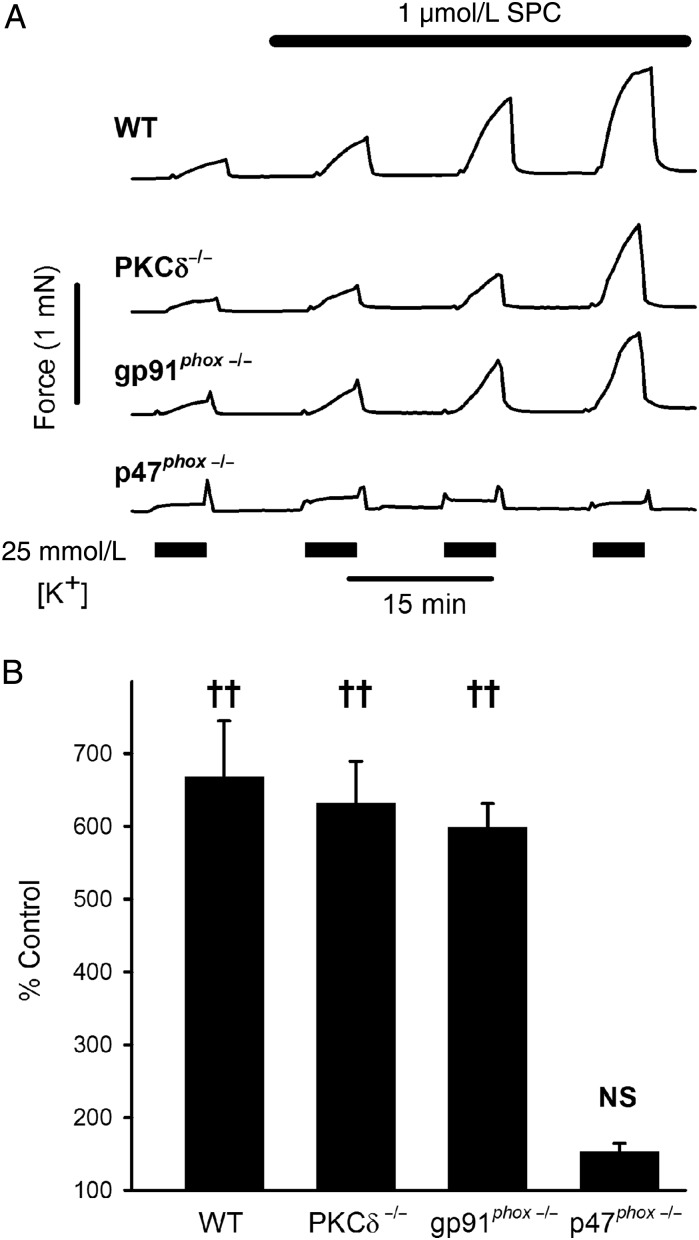
SPC-induced potentiation in MA of mice. We found no difference between MA from C57BL/6 and 129/SV WT mice, so the data were pooled. (*A*) Typical tension recordings from MA of WT, PKCδ^−/−^, gp92*^phox^*^−/−^ and p47*^phox^*^−/−^ mice for 5 min challenges with PSS containing 25 mmol/L [K^+^] in the presence of 1 µmol/L SPC. (*B*) Mean data from WT (*n* = 11, 7 mice), PKCδ^−/−^ (*n* = 4, 4 mice), gp92*^phox^*^−/−^ (*n* = 6, 4 mice), and p47*^phox^*^−/−^ mice (*n* = 11, 7 mice; challenge 2). Bars = SEM. **^††^***P* < 0.001 vs. control; two-way ANOVA, Holm-Sidak *post hoc*.

As previously reported for rat IPA,^10^ 1 µmol/L SPC alone had no effect on tension in rat or mouse MA (e.g. *Figures 1A* and 2*A*), or rat renal or femoral artery.

Rat small MAs were challenged with sequential 5 min applications of PSS containing ∼25 mmol/L [K^+^] to cause a small depolarization-induced rise in tension of 5.8 ± 0.6% (*n* = 31) of that induced by KPSS, as previously described.^10^ SPC (1 µmol/L) was added to the bath, and the procedure repeated at 15 min intervals in the continued presence of SPC. SPC strongly potentiated the subsequent response to depolarization, and this gradually increased with time (*Figure 1A* and *B*), such that at 30 min (second challenge post-SPC) force was increased to 512 ± 40% of control (*n* = 31, *P* < 0.001; *Figure 1C*). Baseline tension measured immediately before each depolarizing challenge was unchanged from control (*Figure 1B*), consistent with the lack of effect of SPC alone, and in the absence of SPC the response to repeated depolarization was also unchanged over 45 min (*Figure 1A*). SPC (1 µmol/L) caused the same degree of potentiation at 30 min in IPA (to 505 ± 39% control, *n* = 47, *P* < 0.001) and small renal arteries (510 ± 107% control, *n* = 9, *P* < 0.01) as MA, but had a smaller effect in large femoral arteries (163 ± 23%, *n* = 12, *P* < 0.05).

SPC also potentiated agonist-induced constriction in MA. Preincubation with SPC (1 µmol/L) caused a substantial leftward shift in the PGF_2α_ cumulative concentration–response curve, reducing the EC_50_ from ∼20 to ∼7 µmol/L (pD_2_: control: 4.78 ± 0.30, *n* = 5; SPC: 5.29 ± 0.14, *n* = 6; *P* < 0.05). We previously reported the same for IPA.^10^

### 3.2 Signalling pathways involved in SPC-mediated potentiation

Arteries were incubated with pharmacological inhibitors for 15 min, and two control depolarizations were performed before 1 µmol/L SPC was added as above. Potentiation at 30 min (second challenge post-SPC) was greatly suppressed or abolished by U73122 (PLC inhibitor, 10 µmol/L), Gö6983 (broad-spectrum PKC inhibitor, 3 µmol/L), and rottlerin (putative PKCδ inhibitor, 1 µmol/L, though see below), but not by Gö6976 (inhibitor of conventional, but not novel PKC, isoforms, 3 µmol/L) or Y27632 (Rho kinase inhibitor, 3 µmol/L; *Figure 1*C). The SPC-induced potentiation of depolarization-induced contraction in MA thus exhibited the same pharmacological profile as we previously reported for IPA,^10^ suggesting the same underlying mechanism.

Based on the differential effects of Gö6976 and broad-spectrum PKC inhibitors, rottlerin, and PKCδ translocation studies, we previously suggested that SPC-induced potentiation of IPA vasoreactivity involved the novel PKCδ isoform.^10^ However, concerns about the specificity of rottlerin^27^ led us to examine this further; indeed, we found no difference between MA from WT and PKCδ^−/−^ mice (*Figure 2A* and *B*).

**Figure 3:**
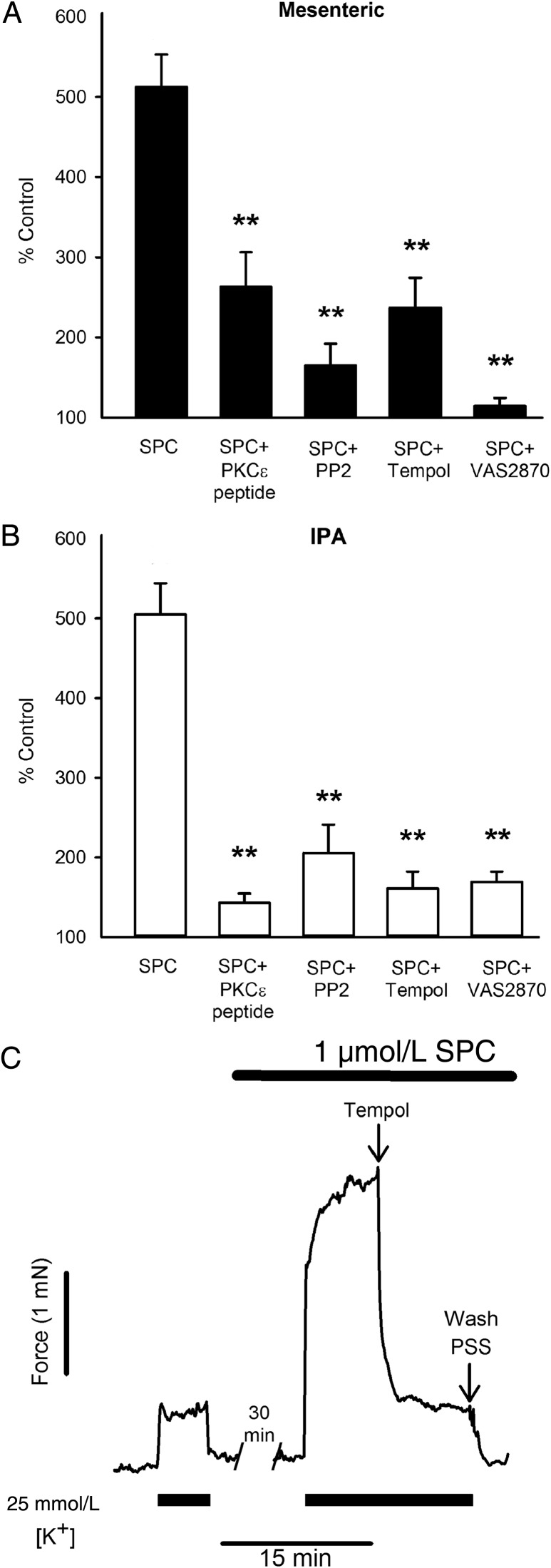
Effects of inhibitors on SPC-induced potentiation in MA and IPA. (*A*) SPC-induced potentiation (challenge 2) of MA for SPC alone (*n* = 31, 24 rats), and in the presence of PKCε peptide inhibitor (*n* = 6), PP2 (Src inhibitor, *n* = 7), Tempol (*n* = 11, 8 rats), and VAS2870 (NOX inhibitor, *n* = 4); 24 rats. (*B*) SPC-induced potentiation (challenge 2) of IPA for SPC alone (*n* = 47, 30 rats), and in the presence of PKCε peptide inhibitor (*n* = 8), PP2 (*n* = 8), Tempol (*n* = 11), and VAS2870 (*n* = 4); 20 rats. (*C*) Typical trace showing reversal of SPC-induced potentiation in a MA by addition of Tempol (3 mmol/L). Bars = SEM. **P < 0.001 vs. SPC alone; two-way ANOVA, Holm-Sidak *post hoc*.

The above precludes any role for PKCδ, suggesting involvement of PKCε, another novel isoform which has been implicated in the actions of SPC.^28^ We were unable to source PKCε^−/−^ mice, but utilized instead the specific PKCε translocation inhibitor peptide (Glu-Ala-Val-Ser-Leu-Lys-Pro-Thr).^29^ This strongly suppressed SPC-induced potentiation of depolarization-induced contraction in both MA and IPA (*Figure 3A* and *B*). SPC (1 µmol/L) also caused translocation of PKCε in cultured PASMCs and MASMCs (see Supplementary material online, *Figure S1*).

### 3.3 Role of NOX and ROS

Both PKCε and Src kinase are known to activate NOX,^30–32^ and Src has been implicated in the actions of SPC in coronary artery.^33^ PP2 (Src inhibitor, 10 µmol/L), VAS2870 (a novel selective inhibitor of NOX, 10 µmol/L),^34,35^ and Tempol (membrane-permeable catalytic superoxide scavenger, 3 mmol/L) all strongly suppressed SPC-induced potentiation of depolarization-induced contraction in both rat MA and IPA (*Figure 3A* and *B*). Similarly, application of Tempol on top of an established constriction reversed SPC-induced potentiation by 95 ± 12% (*P* < 0.01; *n* = 4) in MA (*Figure 3C*).

To determine the NOX isoform, we used the same protocol but with MA from mice lacking gp91*^phox^* (NOX2) or p47*^phox^*, the organizer subunit for NOX2 and NOX1. While potentiation was the same in MA from WT and gp91^phox−/−^ mice, it was absent in mice lacking p47*^phox^* (*Figure 2A* and *B*). This strongly suggests that activation of NOX1 and consequent generation of ROS are essential for the potentiating actions of SPC.

**Figure 4:**
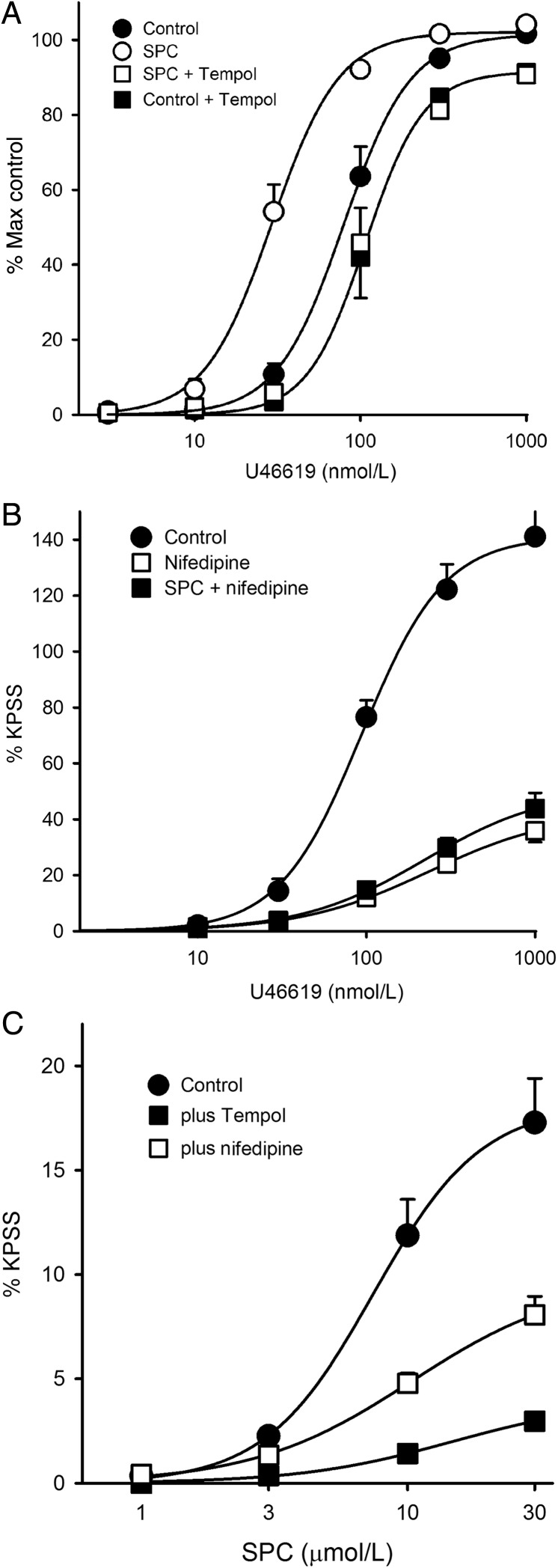
U46619 and SPC-induced contraction, and effects of Tempol and nifedipine. (*A*) Cumulative concentration–response curves for U46619: control (filled circle), SPC (1 µmol/L; open circle), SPC + Tempol (open square); 12 MA (6 rats). Tempol alone (filled square); *n* = 8 MA (4 rats). Mean pD_2_: control: 7.11 ± 0.02; SPC: 7.53 ± 0.03, *P* < 0.001 vs. control; SPC + Tempol: 6.99 ± 0.02, *P* < 0.001 vs. SPC, *P* < 0.02 vs. control; RM ANOVA, Holm-Sidak *post hoc*. pD_2_ for Tempol alone: 6.90 ± 0.08, NS vs. Tempol + SPC; *P* < 0.05 vs. Control; one-way ANOVA, Holm-Sidak *post hoc*. Bars = SEM. (*B*) Similar to *A*, but in the presence of nifedipine (3 µmol/L; open square), and SPC + nifedipine (filled square); 15 MA (9 rats). Control: pD_2_: 7.04 ± 0.06, *V*_max_: 144 ± 13% KPSS; nifedipine: pD_2_: 6.64 ± 0.05, *P* < 0.001 vs. control, *V*_max_: 43 ± 5% KPSS, *P* < 0.001 vs. control; SPC + nifedipine: pD_2_: 6.67 ± 0.03, NS vs. nifedipine alone, *V*_max_: 53 ± 6% KPSS, NS vs. nifedipine alone; RM ANOVA, Holm-Sidak *post hoc*. (*C*) Cumulative concentration–response curves for SPC in MA (filled circles); 16 MA (10 rats), with Tempol (filled squares); 8 MA (5 rats), or nifedipine (open squares); 10 MA (6 rats). SPC: pD_2_: 5.08 ± 0.04, *V*_max_: 16.8 ± 1.3% KPSS; SPC + Tempol: pD_2_: 4.84 ± 0.06, *P* < 0.02 vs. control, *V*_max_: 4.24 ± 1.2% KPSS, *P* < 0.001 vs. control; SPC + nifedipine: pD_2_: 5.10 ± 0.06, NS vs. control, *V*_max_: 8.25 ± 1.1% KPSS, *P* < 0.002 vs. control, *P* < 0.05 vs. Tempol; one-way ANOVA, Holm-Sidak *post hoc*. Bars = SEM (not shown if smaller than symbol).

We examined whether ROS were also responsible for SPC-induced potentiation of agonist-induced contraction. We utilized U46619, as unlike PGF_2α_ it only activates TP receptors; experiments were performed in the presence of 100 µmol/L l-NAME to mitigate against any complicating effects of nitric oxide. U46619 concentration–response curves are repeatable; three were performed on each MA: control, following incubation with SPC (1 µmol/L), and SPC plus Tempol (3 mmol/L). A separate set of time-matched experiments were performed with Tempol alone. SPC caused a large leftward shift in the U46619 concentration–response relationship (*P* < 0.001), which was ablated in the presence of Tempol such that the relationship was shifted significantly to the right of control (*P* < 0.05; *Figure 4A*). There was no difference between the effects of SPC plus Tempol and Tempol alone.

To determine the mechanism by which SPC potentiates U46619-induced contraction, we performed similar experiments using nifedipine (3 µmol/L; L-type channel blocker). Nifedipine suppressed the response to U46619 (*Figure 4B*), and in its presence SPC was without any effect. This suggests that SPC potentiates U46619-induced contraction by enhancing voltage-gated Ca^2+^ entry.

As Tempol suppressed the effects of subcontractile concentrations of SPC, we examined whether it would also suppress the effects of higher concentrations of SPC, which do elicit vasoconstriction. Indeed, Tempol suppressed SPC-induced contraction in MA by ∼75% (*P* < 0.001; *Figure 4C*). Conversely, nifedipine only reduced the response to SPC by ∼50%, as expected because SPC at more than ∼5 µmol/L also activates Rho kinase-mediated Ca^2+^ sensitization.^1,5–9^ These results imply that SPC-induced contraction in MA is largely mediated via ROS.

### 3.4 Does SPC increase ROS generation?

**Figure 5:**
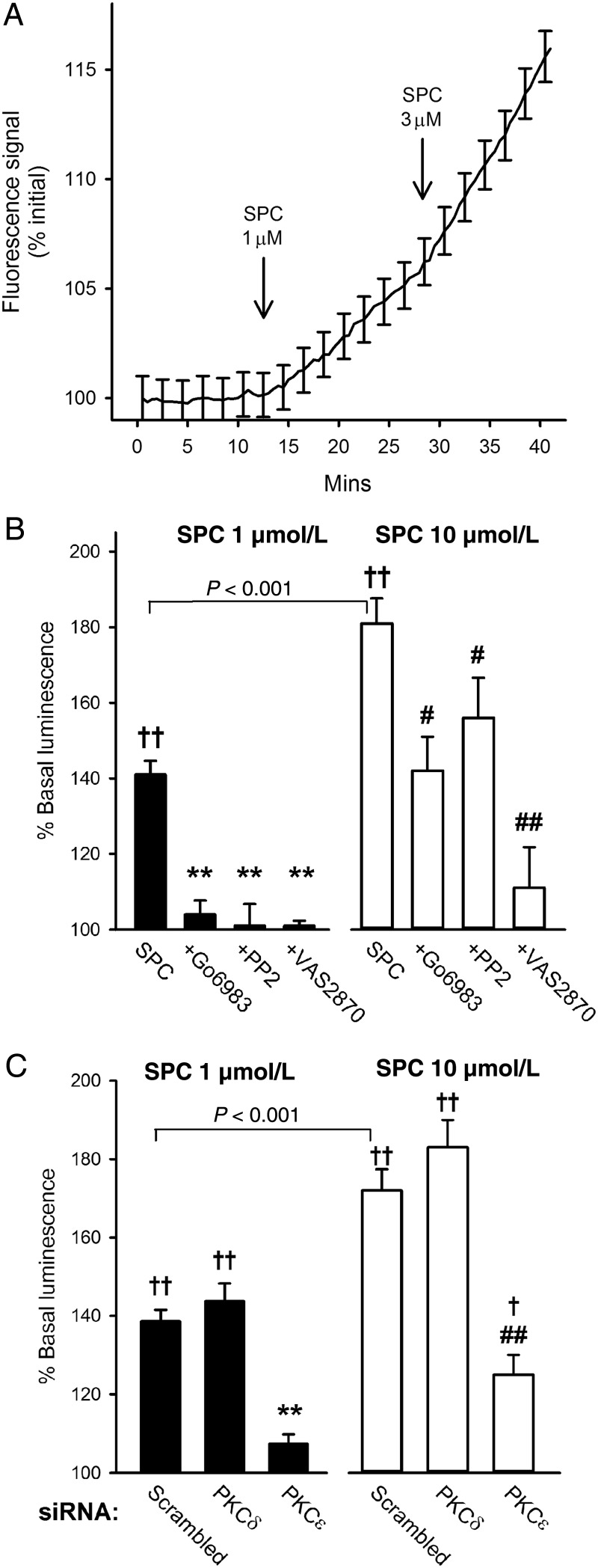
Effect of SPC on ROS generation. (*A*) Mean of real-time recordings from intact MA from three rats showing increased oxidation of C-DCFH (fluorescence at 530 nm) following addition of SPC. Data normalized to initial fluorescence; bars = SEM, shown at 2 min intervals for clarity. (*B*) Increase in lucigenin luminescence in PASMCs cultured from eight rats following addition of 1 (*n* = 11) or 10 (*n* = 12) µmol/L SPC, and in the presence of Gö6983 (*n* = 6 and 7), PP2 (*n* = 9 and 11), and VAS2870 (*n* = 4 and 4). ^†^*P* < 0.01, ^††^*P* < 0.001 vs. control (no SPC); **P < 0.001 vs. 1 µmol/L SPC alone; ^#^*P* < 0.05, ^##^*P* < 0.001 vs. 10 µmol/L SPC alone; one-way ANOVA, Holm-Sidak *post hoc*. Bars = SEM. (*C*) Increase in lucigenin luminescence following addition of 1 or 10 µmol/L SPC in PASMCs (cultured from six rats) transfected with scrambled siRNA (*n* = 12), PKCδ siRNA (*n* = 4), or PKCε-siRNA (*n* = 5). ^†^*P* < 0.01, ^††^*P* < 0.001 vs. control (no SPC); **,^##^*P* < 0.001 vs. scrambled siRNA with 1 or 10 µmol/L SPC; one-way ANOVA, Holm-Sidak *post hoc*. Bars = SEM.

As a qualitative measure of ROS production we examined the effect of SPC on C-DCFH oxidation in intact, unstimulated MA mounted on a myograph. SPC increased the rate of C-DCFH oxidation in a concentration-dependent manner, under identical conditions to those used for contraction studies (*Figure 5A*).

Addition of SPC (1 µmol/L) caused a ∼40% increase in lucigenin-enhanced luminescence in unstimulated cultured PASMCs (*n* = 11, *P* < 0.001), which was effectively abolished by preincubation with Gö6983 (3 µmol/L), PP2 (10 µmol/L), and VAS2870 (10 µmol/L) such that there was no significant elevation above basal luminescence (*Figure 5B*); none of these agents alone had any effect on basal luminescence. SPC (10 µmol/L) had a significantly stronger effect (∼80% increase, *n* = 12, *P* < 0.001, 10 vs. 1 µmol/L SPC). However, while VAS2870 still strongly suppressed the response to 10 µmol/L SPC, Gö6983 and PP2 were only partially effective at this concentration (*Figure 5B*).

To confirm a role for PKCε upstream of ROS generation, and to positively identify the PKC isoform, we transfected cells with siRNA against PKCε or PKCδ, or scrambled siRNA. PKCε-siRNA reduced protein expression to 19.7 ± 2.6% of control, and strongly suppressed the increase in lucigenin luminescence induced by 1 and 10 µmol/L SPC (*n* = 5, *P* < 0.001 for both). PKCδ-siRNA reduced protein expression to 20.5 ± 1.3%, but had no effect on SPC-induced luminescence (*n* = 4; *Figure 5C*). Neither siRNA had a significant influence on basal luminescence.

### 3.5 Do exogenous ROS mimic the effects of SPC?

**Figure 6:**
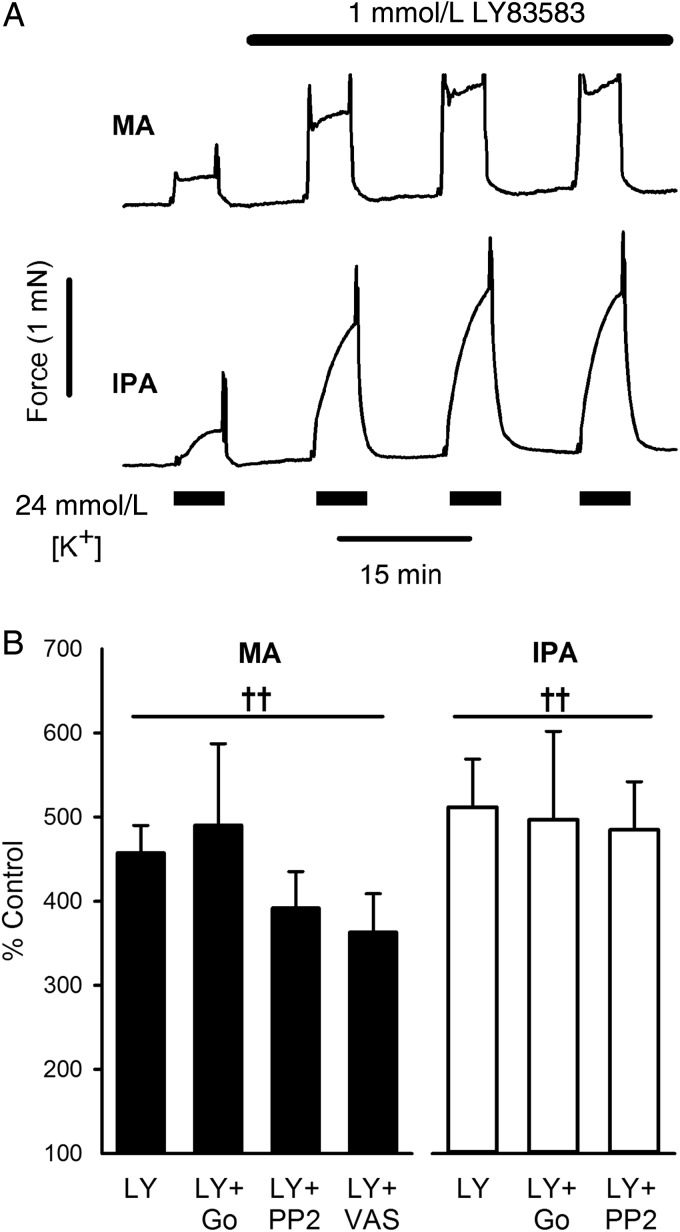
The ROS generator LY83583 mimics the effect of SPC. (*A*) Typical tension recordings from rat MA and IPA for 5 min challenges with PSS containing ∼25 mmol/L [K^+^] and following addition of 1 µmol/L LY83583, showing potentiation of the response. (*B*) LY83583 (LY)-induced potentiation at 30 min in 9 MA (5 rats) and 9 IPA (5 rats), and in the presence of Go6983 (Go, 4 MA and 4 IPA), PP2 (11 MA and 8 IPA), and VAS2870 (VAS, 6 MA); 18 rats. Bars = SEM. ^††^*P* < 0.001 vs. control (no LY83583); no inhibitor had any significant effect; one-way ANOVA, Holm-Sidak *post hoc*.

The above results suggest that SPC-induced potentiation of vasoreactivity is mediated via a PLC-, PKCε- and Src-dependent activation of NOX1, and consequent generation of ROS. We therefore examined whether exogenous ROS could mimic the effects of SPC using LY83583, a membrane-permeable quinolinequinone that acts within the cell to generate intracellular superoxide.^20,36^ At 1 µmol/L, LY83583 does not itself alter vascular tension^20,36^ (and see *Figure 6A*), but like SPC it substantially enhanced the response to depolarization with ∼25 mmol/L [K^+^] in both MA and IPA (*Figure 6A* and *B*; *P* < 0.001). In contrast to SPC, however, the effects were not significantly inhibited by Gö6983, PP2, or VAS2870 (*Figure 6B*), consistent with ROS being downstream of PKCε- and Src-mediated activation of NOX1.

### 3.6 SPC potentiation of voltage-gated Ca^2+^ channels

**Figure 7:**
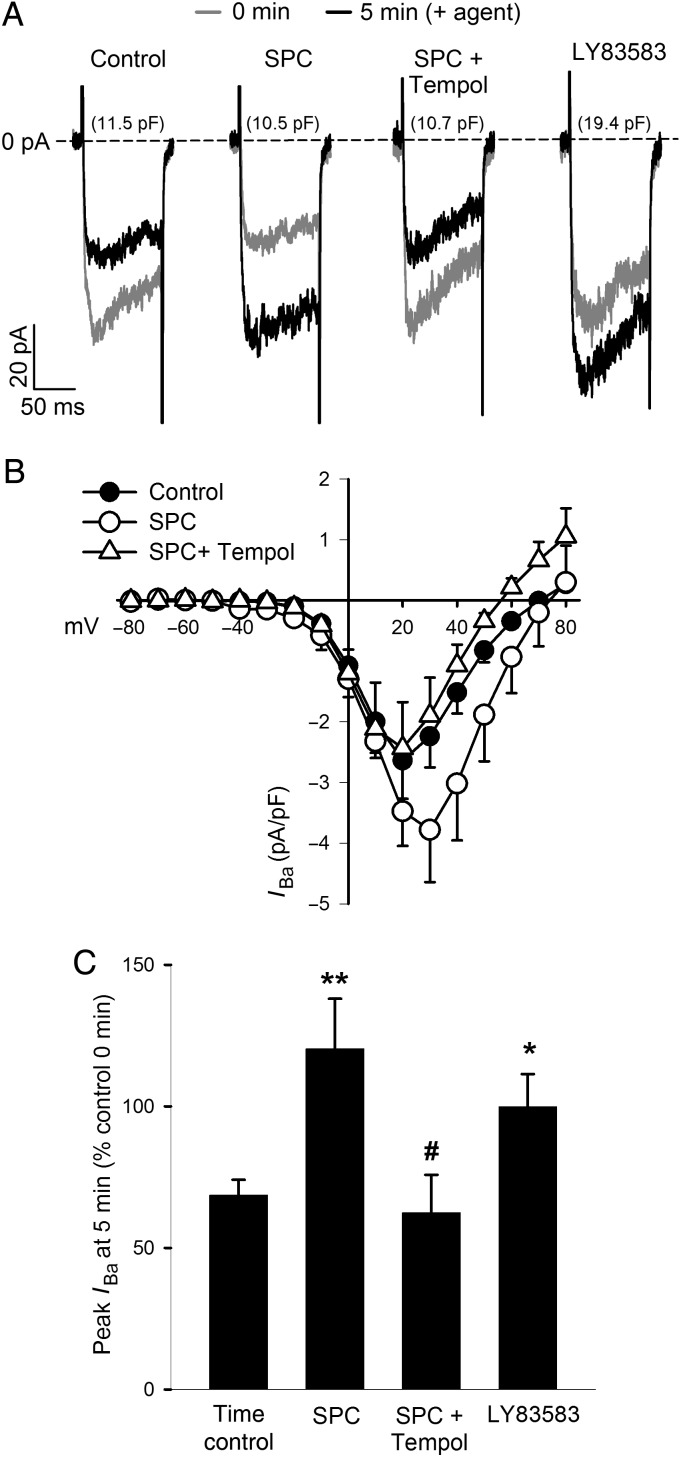
Voltage-gated calcium currents and SPC. (*A*) Representative whole-cell *I*_Ba_ currents following step to +20 mV from holding voltage from four freshly isolated MA myocytes (cell capacitance shown). Grey lines show current immediately following patching (0 min) and before addition of agent; black lines current 5 min later and in the presence of agent. Note current rundown in control trace. (*B*) *I*–*V* plots of *I*_Ba_ densities for MA myocytes from 14 rats (control, closed circles, *n* = 11), in the presence of 200 nmol/L SPC (open circles, *n* = 7), and in the combined presence of SPC and 3 mmol/L Tempol (*n* = 6). Further details in Methods. Bars = SEM. (*C*) Percentage change in peak *I*_Ba_ at 5 min relative to the initial control current at time 0 derived from data above. Time control showing typical *I*_Ba_ rundown, *n* = 11; SPC, *n* = 7; SPC + Tempol, *n* = 6; LY83583 (1 µmol/L, *n* = 6). Bars = SEM. *P < 0.05; **P < 0.001 vs. time control; ^#^*P* < 0.05 vs. SPC alone; one-way ANOVA, Holm-Sidak *post hoc*.

We examined the effects of SPC on voltage-gated Ca^2+^ currents using whole-cell patch-clamp and Ba^2+^ as a charge carrier, in freshly isolated myocytes from rat MA (*Figure 7*). We utilized 200 nmol/L SPC because 1 µmol/L caused rapid loss of attachment. To account for current rundown, comparisons were made between different cells 5 min after addition of SPC or solvent (PSS). SPC (200 nmol/L) increased peak current at 5 min to 176 ± 17% (*n* = 7; *P* < 0.01) of that in control cells, and this enhancement was abolished in the presence of 3 mmol/L Tempol (90 ± 13%, *n* = 6; *P* < 0.01 vs. SPC alone, NS vs. control; *Figure 7*). As predicted, the intracellular ROS generator LY83583 (1 µmol/L) had a similar effect to that of SPC (145 ± 12%, *n* = 6; *P* < 0.05).

## 4. Discussion

We previously reported that concentrations of SPC ≤1 µmol/L, insufficient to elicit elevation of [Ca^2+^]_i_ or vasoconstriction alone, nevertheless strongly potentiate depolarization- and agonist-induced constriction of small IPA by enhancing Ca^2+^ entry.^10^ The precise mechanism, however, remained unclear, and it was unknown whether this phenomenon was limited to pulmonary arteries, which have unique responses to hypoxia and some other stimuli.^20,21,37^ The key findings of the present study are that such concentrations of SPC also strongly potentiate vasoreactivity of small MAs and renal arteries, and that for both MA and IPA this is mediated via an increase in ROS generated by NOX1 and enhancement of Ca^2+^ entry through L-type channels.

SPC (1 µmol/L) enhanced constriction induced by mild depolarization with ∼25 mmol/L [K^+^] by the same extent (∼400% at 30 min) in small IPA, MA, and renal arteries, but to a lesser degree in a large femoral artery (∼60%). The latter may reflect the suggestion that SPC plays a greater role in distal compared with large proximal arteries.^38^ The relatively slow onset, with a maximum effect around 50–60 min (*Figure 1B*), was similar in all artery types and to vasoconstriction induced by high (e.g. 10 µmol/L) concentrations of SPC.^9,33^ While this could reflect accumulation of SPC in the membrane or of a secondary mediator, it is noted that there was no increase in basal tension over at least 45 min (*Figure 1B*). As we previously reported for IPA,^10,20^ preincubation with 1 µmol/L SPC also potentiated the response of MA to PGF_2α_ and U46619, demonstrated here by a large shift to the left of the concentration–response curve (Section 3.1 and *Figure 4A*).

SPC-induced potentiation of vasoreactivity in rat MA exhibited the same pharmacological profile as we reported for IPA^10^ (*Figure 1C*), as it was abolished by the PLC inhibitor U73122 and the putative PKCδ inhibitor rottlerin (but see below), and strongly suppressed by Gö6983 (inhibitor of conventional and novel PKCs), but not by Gö6976 (conventional PKCs only) nor the Rho kinase inhibitor Y27632. PLC has previously been established as a critical component of SPC signalling, and at higher concentrations (>5 µmol/L) SPC strongly activates Rho kinase and Ca^2+^ sensitization in many vascular beds,^1,5,6,9,39^ though the latter is clearly not involved at low concentrations.

The differential effects of Gö6983 and Gö6976 imply a novel isoform of PKC,^29^ which like conventional PKCs are activated by PLC-derived diacylglycerol; conventional PKCs have been previously shown to play no role in the action of SPC.^6,28^ While we originally proposed PKCδ, we show here that SPC-induced potentiation of constriction was unaltered in MA from mice lacking PKCδ (*Figure 2*). However, another novel isoform, PKCε, has been implicated in SPC-induced constriction of cat oesophagus smooth muscle.^28^ Consistent with this, the specific PKCε inhibitory peptide strongly inhibited SPC-induced potentiation in both IPA and MA (*Figure 3A* and *B*).

SPC increases ROS generation in keratinocytes, endothelial cells, and Jurkat cells (albeit all at 10 µmol/L), most likely via NOX,^11–14^ and PKCε is known to activate NOX in cardiac^31^ and pulmonary artery myocytes.^40^ Both PKC and Src phosphorylate the p47^phox^ organizer subunit of NOX1 and NOX2, essential for translocation of cytosolic subunits and activation of the oxidase complex.^32^ Interestingly, Src and PKCε are themselves redox-sensitive, giving rise to the possibility of positive feedback-mediated amplification.^40,41^ Consistent with a central role for NOX-generated ROS, SPC-induced potentiation of depolarization-induced constriction was strongly suppressed in both MA and IPA by the novel NOX inhibitor VAS2870,^34,35^ Src inhibitor PP2, and superoxide scavenger Tempol (*Figure 3*). Tempol also reversed the potentiation by SPC of U46619-induced vasoconstriction (*Figure 4A*). Moreover, the potentiating effect of SPC was abolished in MA from p47^phox −/−^, but not gp91^phox −/−^, mice (*Figure 2*); this strongly implicates NOX1 as the relevant isoform, as only NOX1 and NOX2 require p47^phox^.

Consistent with the above, 1 µmol/L SPC increased ROS generation both in intact MA and in cultured vascular smooth muscle cells, an action effectively abolished in the latter by Gö6983, PP2, and VAS2870, and also following siRNA knockdown of PKCε (but not PKCδ; *Figure 5*). Furthermore, subcontractile concentrations of the intracellular superoxide generator LY83583 mimicked the actions of SPC (*Figure 6*), and we have previously shown that such concentrations also enhance agonist-induced contraction in MA and IPA.^20^ Inhibition of PKC or Src did not affect LY83583-induced potentiation (*Figure 6*), suggesting that in these circumstances PKCε and Src play a primarily upstream role to generation of ROS. These results strongly suggest that the effects of SPC reported here are mediated by increased generation of NOX1-derived ROS.

Concerning the mechanism by which low concentrations of SPC enhance vascular reactivity, our current and previous^10^ results exclude any role for a Rho kinase- (or PKC) mediated increase in Ca^2+^ sensitivity, although at higher concentrations both SPC and ROS do activate Rho kinase.^6,9,20,39^ However, voltage-dependent Ca^2+^ entry induced by depolarization or agonist was increased.^10^ While this might occur if SPC induced some depolarization itself, and NOX-derived ROS have been reported to inhibit K_v_ channels in pulmonary artery,^40,42^ SPC did not suppress K_V_ currents in IPA, and indeed still potentiated the response in maximally depolarized arteries.^10^ Moreover, we have reported that intracellularly generated ROS cause a negative shift in the activation of K_V_ current in both MA and IPA, which would tend to have a hyperpolarizing effect.^20^

ROS and redox state are known to affect the function of L-type Ca^2+^ channels, the α1C subunit of which contains multiple redox-sensitive cysteine residues,^15^ and NOX-derived ROS are reported to enhance L-type Ca^2+^ channel currents in both cardiac and vascular smooth muscle.^16–19^ Consistent with this, we found that 200 nmol/L SPC potentiated L-type Ca^2+^ channel current (Ba^2+^ as a charge carrier) in MA smooth muscle cells, and this was prevented by Tempol and mimicked by the superoxide generator LY83583 (*Figure 7*).

Our results are consistent with a model where subcontractile concentrations of SPC activate NOX1 through a PLC, PKCε, and Src-dependent mechanism, and the consequent increased generation of ROS enhances Ca^2+^ entry through L-type channels, when these are activated by other means. The pathway is apparently identical in MA and IPA. This mechanism may not be limited to SPC, as a similar pathway, albeit at concentrations sufficient to cause constriction alone, has been proposed for the archetypical NOX activator angiotensin II in cerebral arteries,^16^ ET-1 in cardiac myocytes,^19^ and U46619 in pulmonary artery.^17,42^ Notably, Tempol caused a shift to the right of the control U46619 concentration–response curve in MA (*Figure 4A*). While these reports may differ in terms of specific isoforms of PKC and NOX, and in pulmonary artery the possible involvement of K_V_ channels, the underlying signalling pathway is very similar. Note, however, that all these studies utilized agonist concentrations that in themselves cause constriction and activation of parallel Ca^2+^ mobilization and other pathways, which might underlie reported differences.

Considering the above, we briefly examined whether ROS and NOX signalling was important for constriction elicited by higher concentrations of SPC, which has been attributed to activation of voltage-dependent and -independent Ca^2+^ entry and Rho kinase-mediated Ca^2+^ sensitization.^1,6–9^ Notably, all of these have been reported to be activated by ROS.^32,41^ We found that Tempol suppressed SPC-induced constriction by >80% in MA (*Figure 4B*), and that 10 µmol/L SPC doubled the rate of ROS generation compared with 1 µmol/L (*Figure 5B*). While this implies that ROS form a key signalling component for SPC at any concentration, the fact that Gö6983, PP2, and knockdown of PKCε only partially inhibited the elevation of ROS elicited by 10 µmol/L SPC suggests that an additional pathway may be activated by concentrations >1 µmol/L, as previously suggested for other cell types.^43^

## 5. Conclusion

We demonstrate here that low concentrations of SPC, insufficient to elicit vasoconstriction alone, strongly potentiate vasoreactivity via PLC, PKCε, and Src-dependent activation of NOX1, increased generation of ROS, and consequent enhancement of Ca^2+^ entry through L-type channels. SPC has been implicated in cardiovascular disease, though the majority of *in vitro* studies utilized concentrations probably well in excess of physiological levels.^1–3^ The concentrations used here and previously (≤1 µmol/L)^10^ are closer to those reported in plasma,^44^ although physiologically relevant concentrations at the cell surface are difficult to determine because of autocrine and paracrine production, and extensive binding to albumin, HDLs, and LDLs.^1,4,12^ Nevertheless, our results suggest that physiological or pathophysiological concentrations of SPC could greatly increase vascular reactivity to other stimuli. In addition, we can speculate from the data shown in *Figure 4B* that many of the vascular actions of SPC might be mediated via increased ROS. In this respect, there are similarities between SPC and angiotensin II, which share many downstream pathways.^1,45^

The question arises as to why SPC and low concentrations of ROS should have similar effects in MA and IPA,^4,5,9,20^ whereas higher concentrations of exogenous ROS are reported to constrict pulmonary but relax systemic arteries.^20,21,40^ It is probable that this relates to compartmentalization of SPC-induced ROS signalling, synonymous to that for Ca^2+^. Notably, angiotensin II causes highly localized sub-plasmalemmal generation of ROS and co-localized L-type channel activity in cerebral artery, suggesting clustering of receptors, NOX, and L-type channels in a micro-signalling domain.^16^ We speculate that a similar situation exists for SPC and an as yet unidentified high affinity SPC receptor. Higher concentrations of exogenous ROS would, however, have more promiscuous effects, for example relaxing MA (but not IPA) by opening K_v_ channels,^20^ and constricting IPA (but not MA) in part by mobilizing ryanodine-sensitive Ca^2+^ stores.^21,30^

In summary, we present evidence for a novel pathway by which physiological concentrations of SPC strongly potentiate vasoreactivity, involving PLC, NOX1, and ROS-mediated enhancement of voltage-gated Ca^2+^ entry. Similarities with other studies suggest that this could potentially be common to other GqPCR and PLC-coupled agonists, with significant implications for vascular regulation and disease.

## Supplementary material

Supplementary material is available at *Cardiovascular Research* online.

## Funding

This work was supported by the Wellcome Trust (grant #087776). Funding to pay the Open Access publication charges for this article was provided by the Wellcome Trust.
